# Frequency of Extended Red Cell Antigen Phenotype Among Patients of Hematological Diseases: A Single Center Study

**DOI:** 10.7759/cureus.27215

**Published:** 2022-07-24

**Authors:** Faryal Tariq, Rehana Ahmed, Javeria Ashfaq, Warkha Thakur, Asma Ashique, Munira Borhany

**Affiliations:** 1 Haematology, National Institute of Blood Diseases and Bone Marrow Transplantation, Karachi, PAK; 2 Hematology, National Institute of Blood Diseases and Bone Marrow Transplant, Karachi, PAK; 3 Medicine Ward 3, Jinnah Postgraduate Medical Centre, Karachi, PAK; 4 Hematology, National Institute of Blood Diseases and Bone Marrow Transplantation, Karachi, PAK; 5 Cardiology, Tabba Heart Institute, Karachi, PAK

**Keywords:** blood group, antigens, blood transfusions, hematological diseases, alloimmunization

## Abstract

Background

Alloimmunization of erythrocytes is a major problem in patients with hematological diseases that require frequent blood transfusions. Matching of extended red cell antigens of Kell, MNS, Kidd, and Duffy can decrease the risk of alloimmunization. Hence, in this study, the frequencies of the extended red cell phenotypes were explored.

Objective

To find out the frequency of extended red blood cell antigen phenotypes among patients with hematological diseases.

Methods

This cross-sectional research study was performed on 488 patients diagnosed with hematological diseases who required blood transfusion at the National Institute of Blood Disease and Bone Marrow Transplantation Karachi for a period of 1.42 years from November 2019 to March 2021. The blood of patients was analyzed for antigen phenotypes of different blood group systems including Kell, MNS, Kidd, and Duffy. The data obtained were interpreted.

Results

Among the 488 patients, 284 (58.20%) patients were male, and 204 (41.80%) patients were female with a mean age of 8.1 years. Beta thalassemia was the most common hematological disease reported in 354 (72.5%) of the patients. The most common blood group was O positive reported in 182 (37.3%) of the patients followed by B positive blood group in 124 (25.4%). The frequencies of extended red cell antigen phenotypes in the patients were K antigen 14 (2.9%), Kp^a^ antigen 26 (5.3%), Kp^b^ antigen 424 (86.9%), Fy^a^ antigen 360 (73.8%), Fy^b^ antigen 260 (53.3%), Jk^a^ antigen 294 (60.2%), Jk^b^ antigen 326 (66.8%), M antigen 410 (84.0%) and N antigen 306 (62.7%).

Conclusion

Beta thalassemia was the most common hematological disease followed by iron deficiency anemia, aplastic anemia, and acute leukemia. Patients with hematological diseases had a higher prevalence of Kp^b^ antigen followed by M, Fy^a^, Jk^b^, N, Jk^a^, Fy^b^, Kp^a,^ and K antigen. O positive was the most frequent blood group followed by B positive, A positive and AB positive blood group.

## Introduction

Hematologic diseases of the blood and blood-forming organs, afflict millions of people throughout the world [1]. Despite advances in the management of hematologic diseases, these patients often reach a stage where regular blood transfusions are required to maintain their health [2]. Blood transfusion is one of the critical interventions in patients with hematological diseases presenting with symptomatic anemia [3], which requires a compatible and safe transfusion of blood or its components [4]. However, the alloimmunization of red blood cells (RBC) is a major adverse effect of blood transfusion [5]. RBC alloimmunization evolves due to genetic RBC antigen differences between donor and recipient [6].

The International Society of Blood Transfusion (ISBT) has reported 360 erythrocyte antigens. Of these, 322 antigens were distributed in 36 blood group systems, while the remaining 38 were not assigned to any known system [7]. Blood group phenotypes of red cell antigens and their frequencies show variations across different populations in the world [8]. The first and most important system in blood transfusion therapy is the ABO blood group system followed by other blood group systems including Rh, Kell, MNS, Kidd, and Duffy systems. According to ISBT, the MNS blood group system is second, the Kelly system is sixth, the Duffy system is eighth and the Kidd system is ninth in significance among the blood group systems in a clinical hematological intervention [9,10]. In 1927 Karl Landsteiner and Philip Levine described the M and N antigens located on Glycophorin A and B. MNS blood group system genes are controlled by chromosome 4 (4q32.21) and co-dominant alleles pairs (LN and LM). In 1946 Kell blood group system was discovered, and it currently contains more than 30 antigens that indicate the complexity of the Kell system. The most important antigens of the Kell system are K and k antigens. ABO and Rh antigens are highly immunogenic antigens followed by K antigen in third place. The anti-K antibody is associated with significant blood transfusion reactions. In 1950, Marie Cutbush and Patrick Mollison described the Duffy blood group system. Duffy-antigen is also known as Fy glycoprotein, located on the RBC surface. Fya and Fyb antigens are controlled by chromosome 1 (1q23.2). Anti-Fya and Fyb are of IgG type and associated with significant blood transfusion reactions. The Kidd blood group system was described by Beverly Niedziela, Fred Allen, and Louis Diamond in 1951. The antigens Jk are glycoproteins, located on the RBC membrane. Although the formation of anti-Jk antibodies is rare, it can cause severe hemolytic transfusion reactions if alloimmunization occurs [10,11].

Patients with hematological diseases require recurrent blood transfusion, which is considered a major therapy component. Therefore, the provision of phenotypically matched RBCs is most important and challenging for the safe transfusion of blood or its components. Therefore, the current study focuses on finding the frequency of extended red cell antigen phenotypes among patients of hematological diseases registered in our tertiary care institution.

## Materials and methods

The study design was a cross-sectional observational study and was conducted at the National Institute of Blood Diseases and Bone Marrow Transplantation (NIBD & BMT) Karachi. During the study period of 1.42 years from November 2019 to March 2021, 488 patients who presented with anemia and required blood transfusion were selected for this study. The study was granted ethical approval from the Institutional Review Board/Ethics committee NIBD & BMT (Approval #: NIBD/RD-192/03-2019). Informed consent was taken from each patient after full disclosure of the risks and benefits of the study being conducted.

Patients with different hematological diseases including iron deficiency anemia, beta-thalassemia, aplastic anemia, pure red cell aplasia, acute leukemia, sickle cell disease, autoimmune hemolytic anemia, and inherited bone marrow failure syndrome that required blood transfusion for the first time or who had prior transfusions earlier than three months back were included in the study. Patients not requiring blood transfusions or who had transfusions within the past three months were excluded. The sampling was done through an electronic database. There was no age cut-off involved. Any patient regardless of age and gender who satisfied the criteria was included in the study. The sampling was done through electronic records of the patients. The commodities and other information for the patients weren’t relevant to the scope of the study, so were not recorded. Blood samples of each patient suffering from hematological diseases were collected in a sterilized container and serologically tested for blood groups (A, B, AB, and O), Kell system (K, Kpa, and Kpb antigen), Duffy system (Fya and Fyb antigen), Kidd system (Jka and Jkb antigen) and MNS system (M and N antigen). The specimen was appropriately prepared for serological testing according to the manufacturer’s guidelines. The necessary steps regarding the incubation time and other requirements such as temperature etc. were followed according to the manufacturer’s guidelines. After adding the antisera, a positive agglutination reaction was considered as a positive test for the required blood group phenotype. All the negative tests performed through indirect antiglobulin test were confirmed by additionally doing the Coombs test and the data was recorded. The recorded data was then organized, sorted, and entered into a data collection sheet. Data were analyzed and interpreted with SPSS (IBM Corp. Released 2019. IBM SPSS Statistics for Windows, Version 26.0. Armonk, NY: IBM Corp. Descriptive statistics such as percentages and frequencies were computed. The Chi-square test or Fischer exact test was used with a p-value of ≤ 0.05 considered as significant.

## Results

In this study, 488 patients were included, who were registered at our institution. Out of these 488 patients, 284 (58.20%) patients were male, and 204 (41.80%) patients were female with a mean age of 8.1 years.

Beta thalassemia was the most common hematological disease reported in 354 (72.5%) of the patients followed by iron deficiency anemia in 48 (9.8%), aplastic anemia, and acute leukemia in 28 (5.7%) of the patients each. O positive was the most frequent blood group reported in 182 (37.3%) of the patients followed by B positive blood group in 124 (25.4%), A positive blood group in 122 (25.0%), and AB positive blood group in 44 (9.0%) of the patients (Table 1). In the Kell blood group system, K antigen was positive in 14 (2.9%) of the patients, Kpa antigen in 26 (5.3%) patients, and Kpb antigen in 424 (86.9%) patients. In the Duffy blood group system, Fya antigen was positive in 360 (73.8%) patients and Fyb antigen in 260 (53.3%) patients. In the Kidd blood group system, Jka antigen was positive in 294 (60.2%) patients and Jkb antigen in 326 (66.8%) patients. In the MNS blood group system, M antigen was positive in 410 (84.0%) patients and N antigen in 306 (62.7%) patients (Table 1).

**Table 1:** Descriptive Statistics for Hematological Diseases and Blood Group Systems

			Frequency	Percentage
Disease	Beta Thalassemia		354	72.5%
	Iron Deficiency Anemia		48	9.8%
	Aplastic Anemia		28	5.7%
	Sickle Cell Disease		10	2.0%
	Pure Red Cell Aplasia		2	0.4%
	Autoimmune Hemolytic Anemia		2	0.4%
	Acute Leukemia		28	5.7%
	Inherited Bone Marrow Failure Syndrome		16	3.3%
Blood Groups	A +		122	25.0%
	B +		124	25.4%
	O +		182	37.3%
	AB +		44	9.0%
	O -		16	3.3%
	A -		0	0.0%
	AB -		0	0.0%
Kell System	K Antigen	Yes	14	2.9%
		No	474	97.1%
	Kp^a^ Antigen	Yes	26	5.3%
		No	462	94.7%
	Kp^b^ Antigen	Yes	424	86.9%
		No	64	13.1%
Duffy System	Fy^a^ Antigen	Yes	360	73.8%
		No	128	26.2%
	Fy^b^ Antigen	Yes	260	53.3%
		No	228	46.7%
Kidd System	Jk^a^ Antigen	Yes	294	60.2%
		No	194	39.8%
	Jk^b^ Antigen	Yes	326	66.8%
		No	162	33.2%
MNS System	M Antigen	Yes	410	84.0%
		No	78	16.0%
	N Antigen	Yes	306	62.7%
		No	182	36.3%

The antigen frequencies of different blood group systems were compared with hematological diseases. All the blood group systems showed significant association with beta thalassemia at a p-value <0.05. In the Kell blood group system, Kpb antigen was found to be significantly high in beta-thalassemia in 308 (87.0%) of the patients, followed by iron deficiency anemia in 44 (91.6%) patients and aplastic anemia in 27 (6.4%) of the patients while in Duffy blood group system, Fya and Fyb antigens were detected significantly high in beta-thalassemia in 256 (72.3%) and 182 (51.4%) of the patients respectively with a statistically significant relationship (p-value=0.001). K and Kpa antigens were found to be significantly lower in all hematological diseases. However, the Kell blood group system had a statistically significant relationship with Beta Thalassemia, Iron Deficiency Anemia, and Aplastic Anemia as they reported a p-value of <0.001, <0.001, and 0.003 respectively. The frequencies of Kell and Duffy blood group antigens are given in the table below (Tables 2, 3).

**Table 2:** Hematological Diseases and Kell Blood Group System

Diseases	Kell system			P-value
	K (%)	Kp^a^ (%)	Kp^b^ (%)	
Beta Thalassemia	10 (2.8)	16 (4.5)	308 (87.0)	<0.001
Iron Deficiency Anemia	2 (4.1)	2 (4.1)	44 (91.6)	<0.001
Aplastic Anemia	2 (7.1)	3 (10.7)	27 (6.4)	0.003
Sickle Cell Disease	2 (20)	3 (30)	10 (100)	---
Pure Red Cell Aplasia	0 (0)	0 (0)	2 (100)	---
Autoimmune Hemolytic Anemia	0 (0)	0 (0)	2 (100)	---
Acute Leukemia	0 (0)	0 (0)	16 (57.1)	---
Inherited Bone Marrow Failure Syndrome	0 (0)	0 (0)	8 (50)	---

**Table 3:** Hematological Diseases and Duffy Blood Group System

Diseases	Duffy System		P-value
	Fy^a ^(%)	Fy^b ^(%)	
Beta Thalassemia	256 (72.3)	182 (51.4)	0.001
Iron Deficiency Anemia	33 (68.7)	26 (54.1)	0.352
Aplastic Anemia	22 (78.5)	16 (57.1)	0.336
Sickle Cell Disease	10 (100)	7 (70)	0.449
Pure Red Cell Aplasia	2 (100)	1 (50)	---
Autoimmune Hemolytic Anemia	2 (100)	1 (50)	---
Acute Leukemia	16 (57.1)	14 (50)	---
Inherited Bone Marrow Failure Syndrome	8 (50)	8 (50)	---

In the Kidd blood group system, Jka and Jkb antigens were detected significantly high in beta-thalassemia in 204 (57.6%) and 248 (70.0%) of the patients respectively with a statistically significant association (p-value=0.038) while in MNS blood group system, M and N antigens were detected significantly high in beta-thalassemia in 292 (82.4%) and 222 (62.7%) of the patients respectively with a statistically significant association due to a p-value of 0.001. No other significant association was found between the other hematological diseases and Kidd and MNS blood group systems. The frequencies of Kidd and MNS blood group system antigens in comparison to the other hematological diseases are listed below in (Tables 4, 5).

**Table 4:** Hematological Diseases and Kidd Blood Group System

Diseases	Kidd System		P-value
	Jk^a ^(%)	Jk^b ^(%)	
Beta Thalassemia	204 (57.6)	248 (70.0)	0.038
Iron Deficiency Anemia	29 (60.4)	31 (64.5)	0.721
Aplastic Anemia	18 (64.2)	20 (71.4)	0.431
Sickle Cell Disease	9 (90)	9 (90)	1.0
Pure Red Cell Aplasia	1 (50)	2 (100)	1.0
Autoimmune Hemolytic Anemia	1 (50)	1 (50)	---
Acute Leukemia	14 (50)	16 (57.1)	---
Inherited Bone Marrow Failure Syndrome	8 (50)	8 (50)	---

**Table 5:** Hematological Diseases and MNS Blood Group System

Diseases	MNS System		P-value
	M (%)	N (%)	
Beta Thalassemia	292 (82.4)	222 (62.7)	0.001
Iron Deficiency Anemia	43 (89.5)	30 (62.5)	0.740
Aplastic Anemia	26 (92.8)	18 (64.2)	0.360
Sickle Cell Disease	10 (100)	9 (90)	1.0

## Discussion

The current cross-sectional study focused on finding the frequency of extended red blood cell antigen phenotypes among patients with hematological diseases. The availability of information about the frequency of extended red cell antigens in hematological diseases is important for the safe provision of blood transfusion and for avoiding blood transfusion reactions and alloimmunization. Patients with hematological diseases such as beta-thalassemia or anemia are at a higher risk of developing antibodies against blood group antigens other than the ABO system [12]. Practically, it is impossible to match all extended red cell antigens among patients with hematological diseases before a blood transfusion to avoid the risk of alloimmunization due to resources and other constraints. It is also important to note that a lack of knowledge about the frequency of antigens in the relevant population makes blood transfusion difficult, especially in patients who have more than one antigen [13].

In the current study, a total of 488 patients diagnosed with different hematological diseases were analyzed for different blood group systems. The mean age was 8.1 years with a standard deviation of 10.8. In comparison, Boateng et al. reported a median age of nine years in their study conducted for frequencies of red cell phenotypes in patients with sickle cell disease in Ghana [14] while Shin et al. reported that 205 (53.66%) were males and 177 (46.33%) were females in their study conducted in Korea [15].

In a study conducted in the United Arab Emirates, Sajwani et al. reported that O positive was the most common blood group in 29.6% of the patients followed by B positive, A positive, O negative, and AB positive blood group [16]. Similarly, Prinja and Narain in their study conducted in India reported that the most frequent blood group was B, followed by blood groups O, A, and AB are the least prevalent respectively [17]. Comparing these findings with our study, the blood group O positive was the most common blood group found followed by B positive blood group.

Owaidah et al. in a selected population in Saudi Arabia reported that they found a higher frequency of Kpb antigen in their patients [18]. Osaro et al. from Nigeria reported a slightly lower K antigen frequency of 2.0% [19]. In a similar study from Pakistan, Mehmood et al. observed a K antigen frequency of 4.05% [20]. The results are analogous to the findings of our study in which the reported frequency of Kell blood group system antigens were K antigen 2.9%, Kpa antigen 5.3%, and Kpb antigen 86.9%.

In another study conducted by Aranda et al. in a population in Texas USA, low frequencies of Duffy system antigens were reported with Fya being observed in 17.8% and Fyb in 20.7% of the patients [21]. In another study from Pakistan, Jabin et al. reported opposite results i.e., they observed higher frequencies of Fya and Fyb antigens at 58.4% and 39.84% respectively [22]. The observed frequencies of the Duffy system antigens in our study were Fya antigen 73.8% and Fyb antigen 53.3%. Kulkarni et al. in India also reported similar frequencies with Fya antigen higher than Fyb like our study [23].

In the Kidd blood group system, the frequencies of Jka and Jkb antigens in our study were observed to be 60.2% and 66.8% respectively. Shah et al. and Setya et al. reported similar results of the frequencies [24,25]. However, Makarovska-Bojadzieva et al. from the Republic of Macedonia reported different results with low frequencies of both Jka and Jkb at 7.7% and 2.5% respectively [26].

Makroo et al. reported the frequencies MNS system in which they observed higher M antigen followed by N antigen [27]. However, Al-Riyami et al. studied the frequencies in an Omani population and reported a low frequency of M antigen at 48.2% while a very low frequency of 11.0% was reported for N antigen [28]. MNS blood group system antigens in our study were observed with a higher frequency of M antigen at 84.0% followed by N antigen at 62.7%.

The difference in frequency of different antigens was observed among the different populations as compared to our study due to genetic differences among different ethnic groups and due to the selection of a small sample size.

In our study, beta-thalassemia was the most common hematological disease observed in 354 (72.5%) of the patients followed by iron deficiency anemia in 48 (9.8%), aplastic anemia, and acute leukemia in 28 (5.7%) of the patients. Our institute is a tertiary care blood disease-specific institute. And most of the non-severe cases of iron deficiency anemia are treated by general physicians and do not come to this institute unless referred specifically. In our area, patients with iron deficiency anemia are managed with enteral or parenteral nutritional supplements to allow self-recovery and evaluation and management of the cause. Blood is transfused in case of emergency or severely symptomatic anemias. Iron deficiency anemia patients usually do not require multiple transfusion if the cause is eliminated. For these reasons, the number of beta thalassemia patients was higher in our study.

Some of the results of our study were observed to be different from other similar studies conducted around the world. In the beta-thalassemia patients in our study, the K antigen was observed with the lowest frequency of 2.8% as compared to the other antigens. However, in a similar study by Elhence et al., the K antigen was reported in 100% of beta thalassemia patients [29]. Similarly, in this study, the M and N antigens were observed with higher frequencies of 82.4% and 62.7% respectively in beta-thalassemia patients. However, in comparison, El-Beshlawy et al. from Egypt reported much lower frequencies of M and N antigens at 8.14% and 1.16% respectively [30]. Our study results have variations among blood group antigens and different hematological diseases especially in beta-thalassemia due to genetic differences among different ethnic groups. However, most of the studies report the beneficial effects of the extended red cell antigen phenotype on safe blood transfusion.

In our literature search, we found that the studies done on extended red blood cell phenotyping in Pakistan were few. This study adds to that literature and contributes to the knowledge base as well as gives clinicians and blood banks in our community an insight into the prevalence and frequency of these phenotypes in the local population which will further help in safe transfusion practices in patients of hematological diseases.

There were several limitations in our study which are well reflected in the results of our study. Firstly, our study was conducted at a single center, with a small sample size as compared to the large population of Karachi and Pakistan. Secondly, due to the limited availability of healthcare resources, a small number of patients were selected to perform selected extended red cell antigen phenotypes.

## Conclusions

It was concluded that the prevalence of extended red cell antigen phenotype in patients with the hematological disease is essential for compatible and safe transfusion of blood or its components. Beta thalassemia was the most common hematological disease followed by iron deficiency anemia, aplastic anemia, and acute leukemia in our study. Patients with hematological diseases had a higher prevalence of Kpb antigen followed by M, Fya, Jkb, N, Jka, Fyb, Kpa and K antigen. O positive was the most frequent blood group followed by B positive, A positive, and AB positive blood groups.
